# A hydrophobic layer prepared by cyclic grafting of polydimethylsiloxane on magnesium: improved corrosion resistance and biocompatibility

**DOI:** 10.1093/rb/rbac068

**Published:** 2022-09-29

**Authors:** Xiaolong Shen, Hao Zhang, Xin Li, Peichuang Li, Yuancong Zhao, Yunbing Wang, Jin Wang

**Affiliations:** Panzhihua University, Panzhihua 617000, China; Panzhihua University, Panzhihua 617000, China; Third People’s Hospital of Chengdu, Southwest Jiaotong University, Chengdu, Sichuan 610031, China; School of Material Science and Engineering, Southwest Jiaotong University, Chengdu, Sichuan 610031, China; School of Material Science and Engineering, Southwest Jiaotong University, Chengdu, Sichuan 610031, China; National Engineering Research Center for Biomaterials, Sichuan University, Chengdu, Sichuan 610064, China; School of Material Science and Engineering, Southwest Jiaotong University, Chengdu, Sichuan 610031, China

**Keywords:** magnesium, hydrophobic, corrosion resistance, biocompatibility

## Abstract

Magnesium and its alloys have been widely studied as absorbable coronary stent materials. However, the rapid corrosion rate in the intravascular environment inhibits the application of magnesium-based stents. In order to endow magnesium-based stent with appropriate degradation rate and biocompatibility, a hydrophobic layer was constructed by *in situ* cyclic grafting 4,4′-diphenylmethane diisocyanate and aminopropyl-terminated polydimethylsiloxane on pure magnesium. SEM-EDS, X-ray photoelectron spectroscopy and water contact angle were detected to analyze the chemical composition of the layer. The amino groups were confirmed to be introduced on the surface which provide a platform for subsequent modification. The contact angle value of the modified surface is 132.1°, indicating a hydrophilic surface. The electrochemical measurements and immersion tests demonstrated that the hydrophobic layer significantly improved the anti-corrosion ability of the substrate. Besides, the biocompatibility of the hydrophobic surface was examined by platelet adhesion, cytocompatibility *in vitro* and subcutaneous implantation *in vivo*. Immunological and histological results indicated that the hydrophobic layer had excellent biocompatibility. Therefore, the presented study might be a promising method for the surface modification of biomedical magnesium-based stent.

## Graphical Abstract

**Figure:**
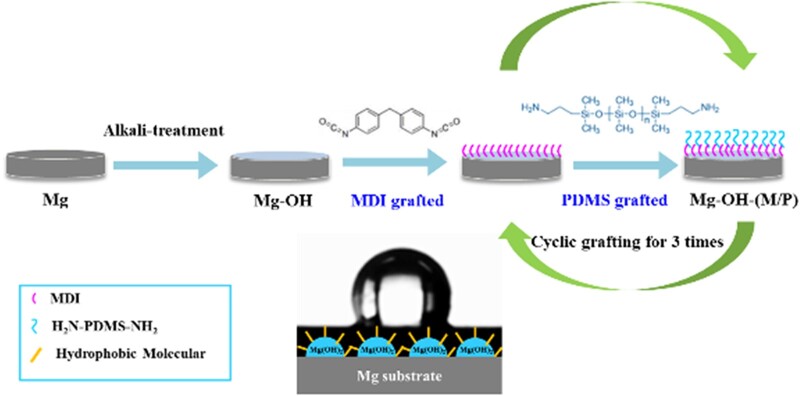

## Introduction

Cardiovascular stent intervention is the most effective method to cardiovascular stenosis. Traditional cardiovascular stents are made of inert materials, like 316 L stainless steel, cobalt–chromium alloys and so on. However, the permanently implanted stents in the blood vessels increase the risks of local inflammation response, in-stent restenosis and late lumen loss [1–4]. Therefore, absorbable coronary stents have attracted increasing attention, which are expected to be gradually degraded after tissue repair [5]. Magnesium-based cardiovascular stents have attracted significant attention of researchers due to the good mechanical properties, biodegradability and biocompatibility [6–8]. An ideal degradable vascular stent should match the process of vascular remodeling in terms of support time and degradation behavior. The vascular remodeling period is generally the first 6 months after stent implantation. During this period, the stent should maintain sufficient mechanical support, which is an important mechanical condition to ensure vascular repair. The stent then gradually degraded at a suitable rate, the degradation process cannot cause adverse reactions in the surrounding tissues, and complete degradation is expected in ∼12–18 months. However, magnesium-based stents are difficult to meet the requirement because of their over-rapid degradation rate in body fluids. Rapid degradation will lead to high local pH value, the enrichment of hydrogen and the premature loss of supporting force, which is unfavorable for tissue repair [9–13].

Therefore, improving the corrosion resistance of magnesium-based stent is the most important problem to be solved. Many surface modification methods have been studied to increase the corrosion resistance of magnesium and its alloys [14–16], including conversion coating [17], micro-arc oxidation [18], electrodeposition [19], fluoride treatment [20, 21], ion implantation [22, 23] and polymer coating [24, 25].

As researches proceed, some researchers begin to fabricate hydrophobic coatings on magnesium to enhance the corrosion resistance since the hydrophobic surfaces can block the close contact of the substrate and the corrosive environment [26–30]. Various methods have been studied for preparing the hydrophobic coatings on magnesium and its alloy, including anodization, micro-arc oxidation, electrodeposition, electrospinning and so on [31–37]. Generally speaking, two key elements need to be possessed for the design of hydrophobic and superhydrophobic surfaces: a rough surface and modification of rough surface with low surface energy molecular [38]. Moreover, the hydrophobic surface can effectively resist platelet adhesion [39–41]. Therefore, constructing a hydrophobic layer on the surface of magnesium-based vascular stents is expected to simultaneously improve corrosion resistance and surface biofunctionalization.

However, the researches of the construction of hydrophobic coatings on magnesium vascular stents are relatively rare. Most of preparation methods are complicated and contain less environmentally friendly chemical reagents. At the same time, the preparation methods of most hydrophobic coatings are complex, expensive, contain some harmful reagents or cannot be prepared on complex surfaces. In addition, whether the surface of the hydrophobic layer has the ability of secondary grafting of biomolecules is crucial. If the surface of the hydrophobic layer has specific functional groups, such as carboxyl groups, amino groups, etc., functional biomolecules can be effectively grafted to further endow the surface with biological functions [42–44].

In this study, the pure magnesium is first treated with alkaline to form a rough microstructure surface, then the 4,4′ diphenylmethane diisocyanate (MDI) and aminopropyl-terminated polydimethylsiloxane (NH_2_-PDMS-NH_2_) are cyclic grafted alternately on the surface of alkaline pretreated magnesium to obtain a hydrophobic layer. The MDI is a kind of organic silicon. Organic silicon has unique material characteristics, such as hydrophobicity, low surface energy and good biocompatibility, so it has been widely used in medicine. The PDMS has strong hydrophobicity and low surface tension, so it is commonly used for the construction of hydrophobic surface [45, 46]. In addition, the PDMS has been widely used to make microfluidic chips for cell culture due to its good biocompatibility [47, 48].

The hydrophobic surface could block the contact between the substrate and corrosive environment, which was expected to contribute to the improvement of corrosion resistance. Furthermore, the hydrophobic layer has good biocompatibility, and the layer could provide amino groups for subsequent grafting of biomolecules. The corrosion resistance and biocompatibility of the hydrophobic layer were investigated systematically.

## Materials and methods

### Materials

The pure magnesium ingot of 99.99% purity was purchased from Zhongxin Metal Co., Ltd., China. It was cut into disks with the diameter of 10 mm and a thickness of 1.2 mm before use. MDI with a molecular weight of 250 and NH_2_-PDMS-NH_2_ with a molecular weight of 1000 were purchased from Sigma (Sigma-Aldrich Chemical Co.). The phosphate buffered saline (PBS) solution with pH = 7.4 was used for degradation tests *in vitro*.

### Fabrication of the samples

**Figure 1.**
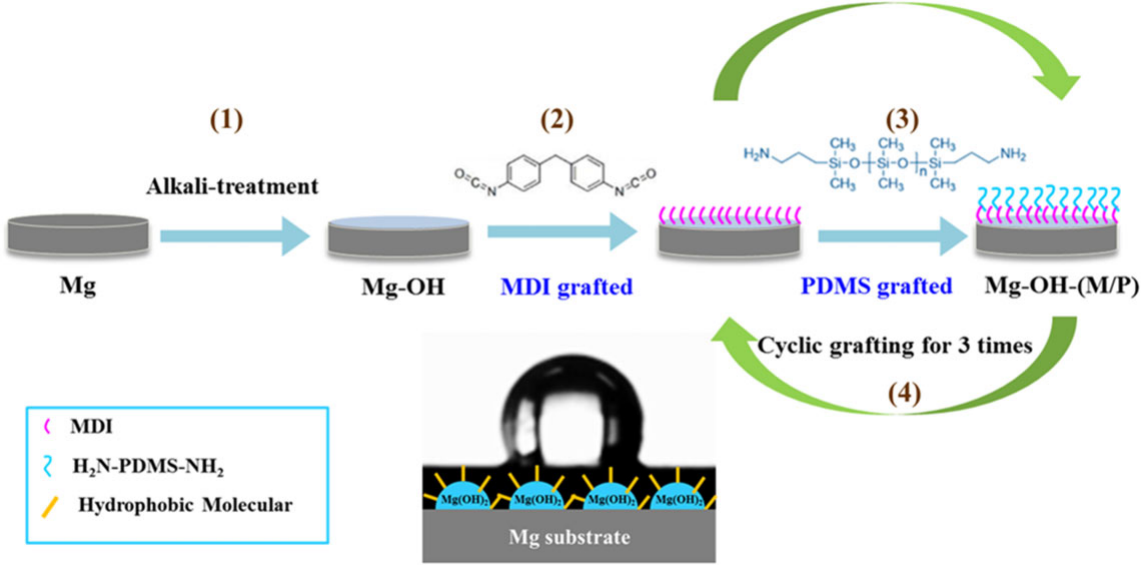
The schematic diagrams of cyclic grafting of MDI and PDMS on the magnesium surface. (1) Alkali-treatment, 4 mol/l, 4 h, 75°C; (2) 2 mg/ml MDI, 60°C, 30 min; (3) 2 mg/ml PDMS, 60°C, 30 min; (4) cyclic grafted for three times.

The schematic diagram of the surface modification is displayed in Fig. 1. Pure magnesium disks were first grinded with SiC paper (from 320 to 5000 grits), subsequently rinsed with deionized water and ethanol for three times separately and dried in N_2_ at last. The prepared pure magnesium specimens were marked as Mg. After that, the samples were immersed into 4 mol/l NaOH solution for 4 h at 75°C and then cleaned and dried, which was marked as Mg-OH. After alkali treatment, the Mg-OH samples were put into the reactor immediately, 2 mg/ml MDI solution (dimethylacetamide (DMAC) is the solvent) was added into the reactor at 60°C for grafting of MDI on the Mg-OH. After reacting for 30 min, the MDI solution was aspirated, and the samples were washed with DMAC. Subsequently, 2 mg/ml NH_2_-PDMS-NH_2_ solution (tetrahydrofuran (THF) is the solvent) was added into the reactor for 30 min at 60°C for grafting of NH_2_-PDMS-NH_2_. After that, the solution was drawn out and THF was added into the reactor for washing. This completed the grafting of the first layer. In this way, three cycles of grafting were carried out on the surface of the sample, and the obtained sample was marked as Mg-OH-(M/P).

### Surface characterization

The surface morphologies of the samples were analyzed by a field emission scanning electron microscope (JSM-7401F, JEOL, Japan). The surface chemical compositions and high resolution spectra of Mg 1 s, O 1 s, C 1 s, N 1 s and Si 2p were analyzed by X-ray photoelectron spectroscopy (XPS, XSAM800, Kratos Ltd, UK). The water contact angle was measured by DSA100 contact angle goniometer (KRUSS, Germany) at room temperature and under standard atmospheric pressure, the tests were measured no less than six times to obtain the average value. The concentration of amine groups on the surface was detected by the acid orange II (AOII) method [49].

### Electrochemical corrosion behavior

The potentiodynamic polarization (PDP) and electrochemical impedance spectroscopy (EIS) in the PBS solution at 37°C were tested to analyze the electrochemical corrosion behavior of samples. The tests were carried out at an electrochemical workstation (IM6, Zahner, Germany). All the samples were molded in epoxy resin with one exposed side as the working electrode, using Cu wire as the conducting wire. The saturated calomel electrode was performed as the reference electrode and a platinum electrode was performed as the counter electrode. The working electrode was first immersed into the PBS solution for 15 min to stabilize the open circuit potential. The PDP curves were subsequently scanned from −2 V to −1 V with the scanning rate of 1 mV/s. The corrosion potential (E_corr_) and corrosion current density (i_corr_) of all the samples were obtained by the Tafel extrapolation. The EIS measurements were tested on the same workstation. The samples were first immersed in PBS solution for 30 min, then tested at the open-circuit potential in a frequency range from 10^−2^ to 10^5^ Hz. All the tests were repeated no less than three times. The representative data of parallel samples were selected for drawing electrochemical impedance spectrum.

### Immersion degradation behavior

Degradation tests *in vitro* were carried out in PBS solution (pH = 7.4) for 210 h at 37 ± 0.5°C. Before testing, the samples were first encapsulated in epoxy resin with an area of 0.785 cm^2^ exposed to the PBS solution. The hydrogen gas volume and pH value of the solution were detected at different intervals during the immersion process.

### Hemocompatibility

Bovine serum albumin (BSA) adsorption and platelet adhesion tests were detected as described previously [50]. The constructed layer contains hydrophobic PDMS molecular chains, which have a strong adsorption effect on albumin. The micro-BCA protein assay kit, purchased from Thermo Fisher Scientific, was used to detect the amount of adsorbed protein on the surface; 10 mg/ml BSA solution was prepared and spread over the sample surface evenly. Then the samples were incubated in 37°C for 30 min and washed with PBS solution. Mix the A, B and C solutions in the micro-BCA kit at a ratio of 25:24:1, drop 0.15 ml mixed solution on each sample and reaction at 37°C for 15 min. The absorbance value of the reacted solution was detected at 562 nm UV wavelength. The protein content on the surface was calculated according to the standard curve. The platelet-rich plasma (PRP) was collected by centrifugation of fresh whole blood at 1500 r/min for 15 min; 150 μl PRP was added to each sample surface and incubated at 37°C for 30 min. After that, the samples were washed with NaCl solution for three times and fixed by 2.5% glutaraldehyde for 6 h. Then the samples were dealcoholized for SEM observation to evaluate the adhesion and activation of the platelets on the samples. Hemolysis ratio was determined according to ASTM F756-00. Hemocompatibility tests were carried out in compliance with the ethics rules.

### Vascular cell growth

Human umbilical vein endothelial cells (ECs) and vascular smooth muscle cells (SMCs) were used to evaluate the vascular cell growth on the samples *in vitro*. The samples were UV-sterilized and placed into cell culture plates. Then the cells were seeded into the plates with samples and incubated in a standard cell culture incubator. After 2 h and 1 day, the samples were removed and the viability of cells on the plates (indirect contact with the sample) were evaluated by cell count and Cell Counting Kit-8 (CCK 8). The cells were stained by Rhodamine 123 and observed by fluorescence microscope. The details of the experimental procedures were carried out as described previously [51].

### *In vivo* subcutaneous implantation

To evaluate the corrosion behavior of Mg, Mg-OH and Mg-OH-(M/P) *in vivo*, four healthy rats (each body weighing ∼ 1.5 kg) were used for subcutaneous implantation. All procedures are in accordance with the Chinese Society for the Protection of Animals. After anesthetizing the rats with pentobarbital sodium (30 mg ml^−1^, ∼1 ml for each rat), Mg, Mg-OH and Mg-OH-(M/P) disks were subcutaneously implanted on both sides of the back. The samples with surrounding tissues were collected to evaluate the degradation and tissue responses after implanted for 30 days. The harvested samples were cleaned by the chromic acid solution to clean the corrosion products on the surface, then they were weighed to measure the remaining weight of the samples. Moreover, the surface morphologies of the cleaned metal samples were characterized using an optical microscope. The tissues around the samples were fixed in paraformaldehyde for 3 days, then embedded in paraffin for tissue sections and stained with hematoxylin and eosin staining for further investigation.

### Statistical analysis

All the tests were carried out at least three times for statistical analysis. The results were expressed as mean value ± standard deviation. The statistical analysis was employed by one-way ANOVA analysis, and the *P* values < 0.05 was considered as significant differences.

## Results and discussion

### Surface characterization

**Figure 2.**
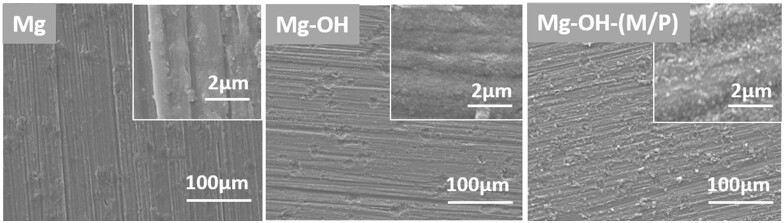
The surface morphologies of the Mg, Mg-OH and Mg-OH-(M/P).

The SEM images of the bare Mg substrate, Mg-OH and Mg-OH-(M/P) are shown in Fig. 2. The bare Mg shows a relatively uniform surface with tiny grooves, which was created by the polish. After alkali-heat treatment, the surface of Mg-OH displays a smoother surface than Mg, which was caused by the formation of the Mg(OH)_2_ on the surface. It is noteworthy that after grafting MDI and NH_2_-PDMS-NH_2_, tiny particles appeared on the surface of Mg-OH-(M/P). This may be due to the surface grafting of molecules. The surface chemical properties need to be further investigated.

**Figure 3.**
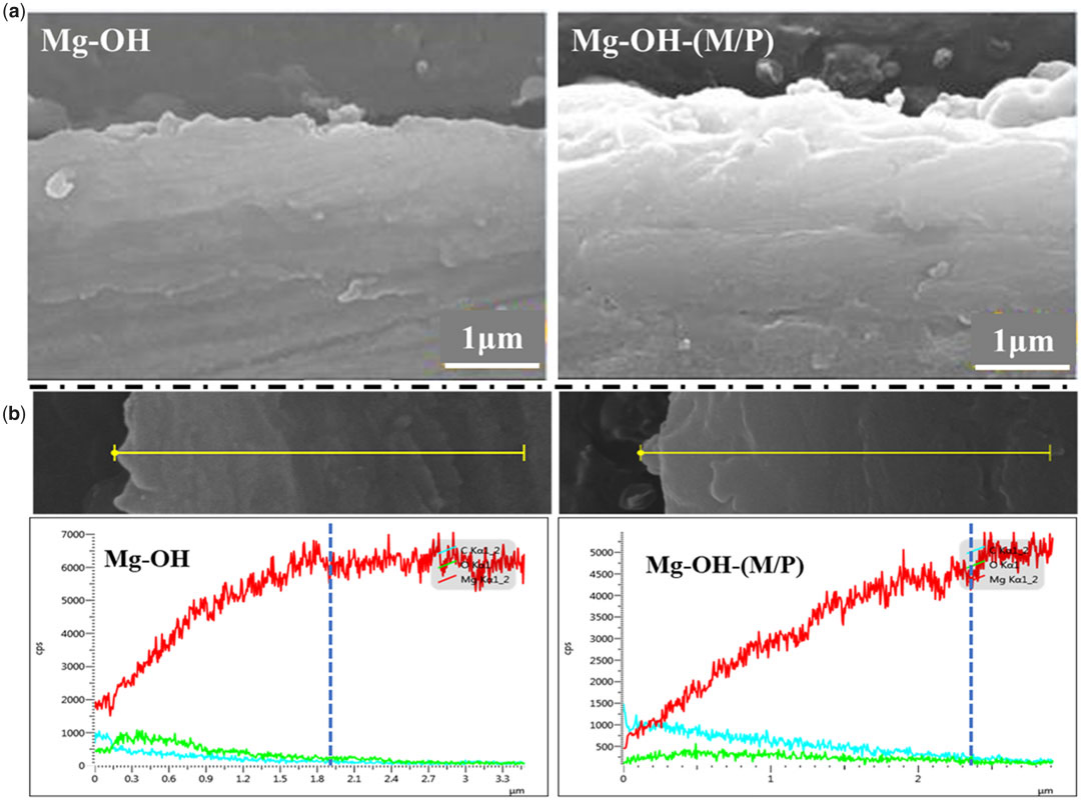
The cross-section SEM images (**a**) and EDS line analysis (**b**) of Mg-OH and Mg-OH-(M/P).

The cross-section morphologies and element distribution of the layer were analyzed using SEM and EDS line scanning, and the results are shown in Fig. 3. As shown in Fig. 3a, neither Mg-OH nor Mg-OH-(M/P) showed a distinct layer–substrate interface. This may be because the magnesium hydroxide layer was gradually grown through the continuous oxidation of the magnesium substrate. This also reflects that the coating may have good adhesion. Through the element distribution of the cross section in Fig. 3b, the thickness of the layer can be inferred. The Mg content of Mg-OH and Mg-OH-(M/P) both gradually increased from the surface to the substrate; in comparison, the C and O content showed a slight decrease. In addition, the content of C element on the surface of Mg-OH-(M/P) was obviously more than that of Mg-OH, which was due to the hydrophobic layer on the surface. The results showed that the thicknesses of the Mg-OH and Mg-OH-(M/P) surface layer were ∼ 1.9 μm and 2.4 μm, respectively. However, the N and Si element in the hydrophobic layer was not detected on Mg-OH-(M/P), which may be due to the low content of these two elements in the layer. Further material characterization is required.

**Figure 4.**
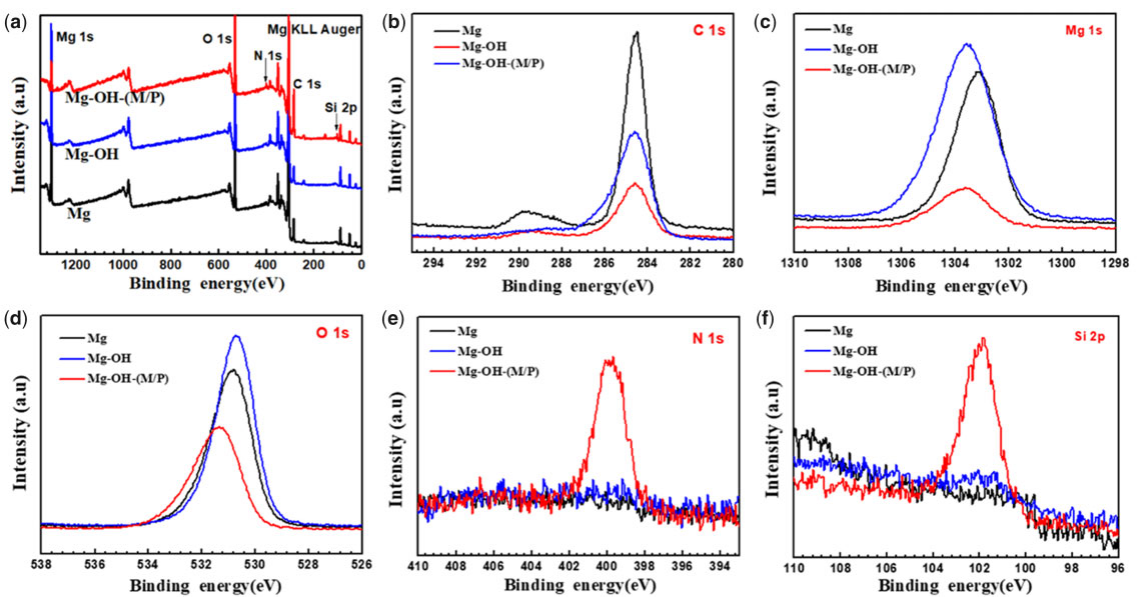
(**a**) XPS wide scans; (**b**) C 1 s high-resolution spectra; (**c**) Mg 1 s high-resolution spectra; (**d**) O 1 s high-resolution spectra; (**e**) N 1 s high-resolution spectra and (**f**) Si 2p high-resolution spectra of the Mg, Mg-OH and Mg-OH-(M/P).

XPS was performed to further measure the chemical states of surface elements on the samples. As shown in Fig. 4 and Table 1, the enhancement of O 1 s peak of Mg-OH was due to the alkali treatment. For Mg substrate, the concentration of O 1 s (52.65%) demonstrated that a MgO film formed by natural oxidation. After the alkali treatment, the concentration of O 1 s increased and the Mg 1 s decreased, which indicated that a new oxide coating has been formed on the surface. After the cyclic grafted of MDI and PDMS for three times, the Mg-OH-(M/P) showed the peak of N 1 s and Si 2p, indicating the MDI and PDMS molecule were immobilized on the surface successfully.

**Table 1.** The surface element concentration of different samples

Sample	Atomic concentration (at.%)				
	Mg	O	C	Si	N
Mg	35.01	52.65	12.35	–	–
Mg-OH	27.06	57.64	15.29	–	–
Mg-OH-(M/P)	7.78	45.24	38.47	4.5	4.01

**Figure 5.**
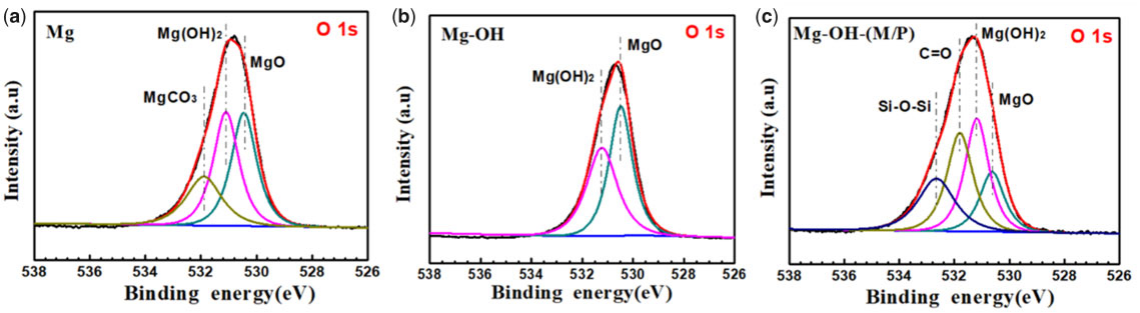
Curve-fitting results of XPS high-resolution O 1 s of the (**a**) Mg, (**b**) Mg-OH and (**c**) Mg-OH-(M/P).

Further investigation of O 1 s peak fittings were performed (Fig. 5). The O 1 s peak of the Mg could be resolved into three typical peaks at 531.9 eV (magnesium carboxylates), 531.2 eV (Mg(OH)_2_) and 530.4 eV (MgO) [49, 50]. After alkali treatment, the O 1 s peak of the Mg-OH could be curve fitted into 531.2 eV (Mg(OH)_2_) and 530.4 eV (MgO), indicating the new formed oxide layer. After the graft of MDI and PDMS, the O 1 s peak could be fitted into four typical peaks at 532.6 eV (Si-O-Si from PDMS), 531.6 eV (C = O from MDI), 531.2 eV (Mg(OH)_2_) and 530.4 eV (MgO). Taking the above results into consideration, one can conclude that the MDI and PDMS were immobilized on the surface successfully.

**Figure 6.**
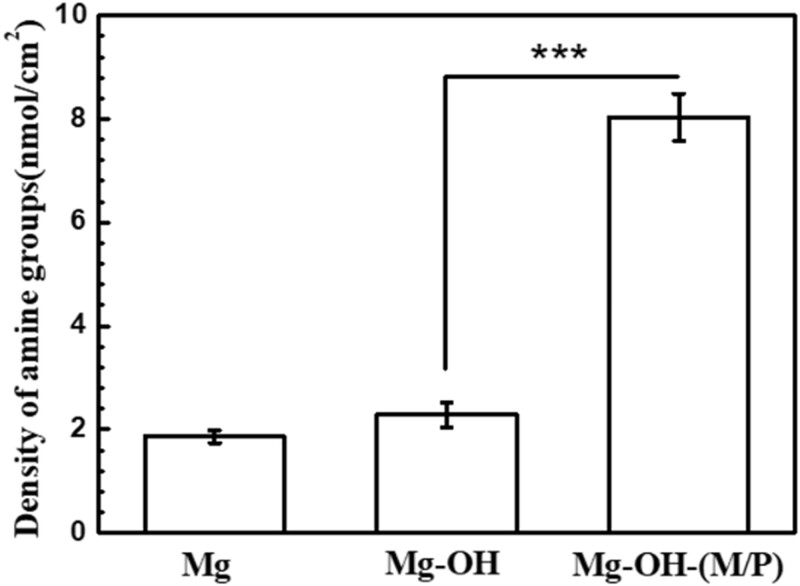
–NH_2_ density on the surface of Mg, Mg-OH and Mg-OH-(M/P).

The amine groups on Mg-OH-(M/P) can be used to immobilize biomolecules to further improve the biocompatibility. Therefore, the density of amine groups on samples surfaces was detected by AOII colorimetric method and shown in Fig. 6. The Mg-OH-(M/P) possessed a maximum value of ∼8.03 nmol/cm^2^. The amine density of 0.08 and 0.1 nmol/cm^2^ was detected on the Mg and Mg-OH. This is due to the physical adsorption of acid orange by the oxide layer on the surface Mg and Mg-OH. The amine groups on the Mg-OH-(M/P) can be used to graft biomolecules for further modification.

### Surface wettability

**Figure 7.**
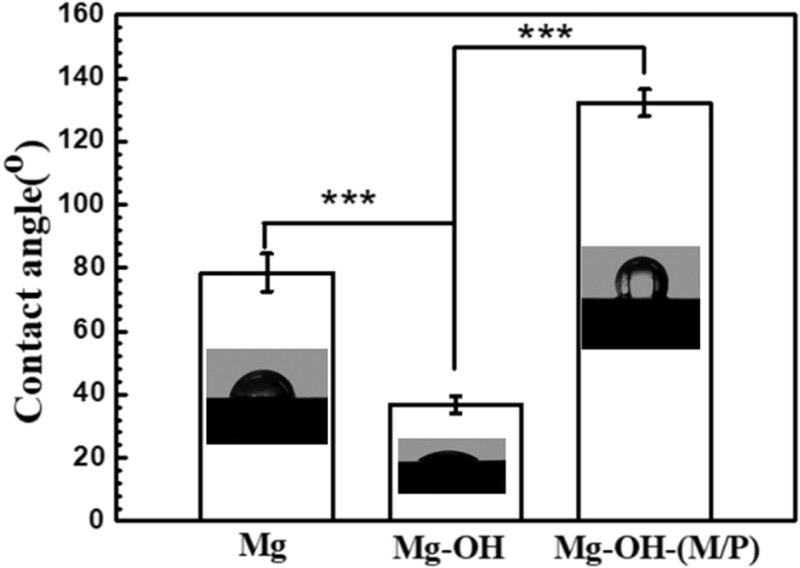
Variation in the water contact angles of Mg, Mg-OH and Mg-OH-(M/P).

The wettability of the coatings on magnesium is very important for the corrosion resistance. Recently, studies have shown that constructing hydrophobic surfaces on magnesium alloys is an effective method of to enhance their corrosion resistance [24–28]. The hydrophobic surfaces have the ability to hinder the close contact of a surface with the corrosive environments. The water contact angles of Mg, Mg-OH and Mg-OH-(M/P) are measured to investigate the surface wettability. As shown in Fig. 7, the bare Mg surface showed a water contact angle of ca. 78.5°; however, the ca. of Mg-OH apparently decreased to 36.7°. The Mg-OH had a large amount of –OH groups on the surface which introduced by the alkali treatment. It is worth noting that, after cyclic grafted of MDI and PDMS, the Mg-OH-(M/P) showed a contact angel value of 132.1°, indicating a hydrophilic surface. The corrosion resistance of this hydrophilic surface requires further study.

### Electrochemical corrosion behaviors

**Figure 8.**
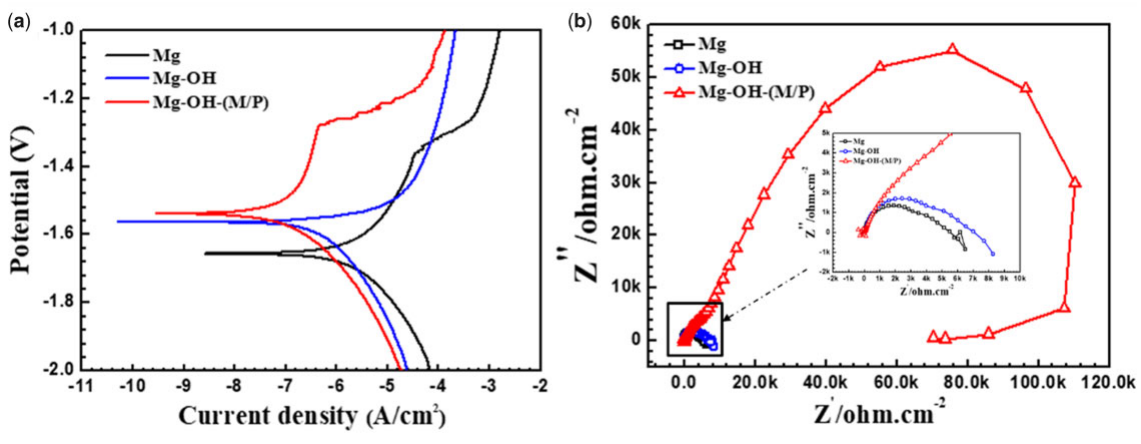
(**a**) The PDP analysis and (**b**) EIS spectra of Mg, Mg-OH and Mg-OH-(M/P).

The corrosion properties of all the samples were first characterized by the PDP curve, shown in Fig. 8a. The corresponding corrosion potential and current values determined by the Tafel method are listed in Table 2. The E_corr_ of Mg-OH and Mg-OH-(M/P) showed a significant positive shift compared to that of Mg. The passive layer of Mg(OH)_2_ was formed on the surface of pure magnesium after the alkali heating treatment, which lead to the improved corrosion resistance. Furthermore, the i_corr_ of Mg-OH-(M/P) was the lowest among all the samples, about two orders of magnitude lower than the bare Mg, which indicated the effectiveness of the hydrophobic surface to improve the corrosion resistance of the substrate. Figure 8b showed the Nyquist plots of the samples in PBS, the Mg-OH-(M/P) showed a remarkable larger impedance than the Mg and Mg-OH, which indicated a higher corrosion resistance.

**Table 2.** The corrosion potentials E_corr_, the corrosion current densities i_corr_ and the corrosion rate Pi of the samples in PBS solution at 37 ± 0.5°C

Samples	E_corr_/V	log i_corr_/A cm^−2^	P_i_ (mm/year)
Mg	–1.65	–5.83	0.033797
Mg-OH	–1.56	–6.36	0.009744
Mg-OH-(M/P)	–1.53	–7.14	0.001655

### Immersion degradation

**Figure 9.**
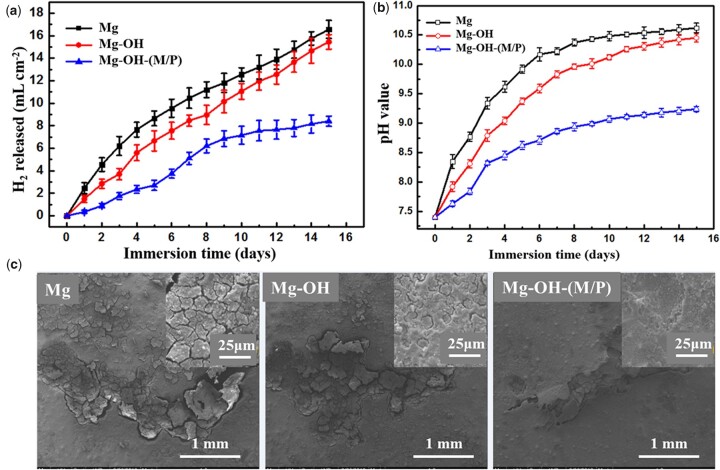
Immersion degradation results of the samples in the PBS solution at 37 ± 0.5°C for 15 days: (**a**) volume of hydrogen evolution; (**b**) pH value; (**c**) SEM images after immersion.

According to the corrosion reaction of magnesium, Mg + 2H_2_O → Mg^2+^ +2OH^−^ + H_2_, the hydrogen evolution and the increased pH of the solution reflect the corrosion rate indirectly. The hydrogen evolution (Fig. 9a) and pH value (Fig. 9b) of Mg, Mg-OH and Mg-OH-(M/P) were detected as a function of immersion time in PBS at 37°C. The SEM images after immersion of 15 days are also shown in Fig. 9c. The volume of H_2_ released from Mg was the most, ∼ 17 ml cm^−2^, and the release rate was the fastest. The Mg-OH released ∼ 15 ml cm^−2^ H_2_ during the immersion, and the release rate is lower than that of Mg in the early stage due to the oxide layer on Mg-OH. However, the oxide layer is chemically unstable, the corrosion rate accelerated after the layer destroyed. The Mg-OH-(M/P) showed a noticeably lower evolved H_2_ volume (∼ 7 ml cm^−2^), with lower H_2_ evolution rate than that of Mg and Mg-OH. This can be attributed to the hydrophobic layer reduced the contact area of substrate and the corrosive environment. The similar trend was observed in pH change. The Mg-OH-(M/P) exhibited the least increase of pH, as compared with Mg and Mg-OH. SEM images after immersion of 15 days further revealed that Mg-OH-(M/P) retained a relatively smooth and complete coverage, while the Mg and Mg-OH underwent a more severe corrosion with network of cracks.

### Blood compatibility

**Figure 10.**
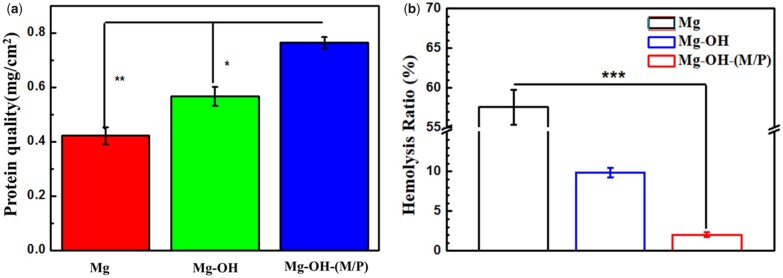
(**a**) BSA adsorption and (**b**) hemolysis ratio of Mg, Mg-OH and Mg-OH-(M/P). Data are presented as mean ± SD and analyzed using a one-way ANOVA, *P < 0.05, **P < 0.01, ***P < 0.001.

Studies have shown that the immobilization of albumin on the surface of the material can endow it with anticoagulant function. Therefore, studying the adsorption capacity of albumin on the surface of the sample can reflect the anticoagulant capacity of the sample. To determine the adhesion of albumin on sample surface, BSA was used in the test via micro-BCA protein assay kit. As shown in Fig. 10a, the Mg-OH-(M/P) adsorbed most amount of BSA (0.76 mg/cm^2^). A small amount of Mg(OH)_2_ and MgO were formed on the Mg sample due to oxidation, which showed protein adsorption (0.42 mg/cm^2^). The oxide layer formed on the Mg-OH is loose and porous, and it is easier to adsorb water and protein than Mg, so the BSA adsorption capacity is more, which is 0.57 mg/cm^2^. Due to its strong hydrophobicity, the surface of Mg-OH-(M/P) is prone to irreversible adsorption with proteins, so the Mg-OH-(M/P) adsorbed the largest amount of BSA.

Since hemocompatibility is important for blood-contacting materials, the hemolysis ratio and plate adhesion assay were carried out. Hemolysis rate below 5% is generally acceptable. The hemolysis rates of the samples are shown in Fig. 10b. The bare Mg exhibited the highest hemolysis rate (∼57%). This could be due to the fast degradation rate of bare magnesium, leading to excessively high osmotic pressure and pH in the local environment, which caused hemoglobin to rupture, and ultimately led to a high hemolysis rate. The hemolysis rate of Mg-OH decreased obviously, the Mg(OH)_2_ layer formed by alkali treatment inhibited the corrosion of the substrate to some extent. However, the hemolysis rate of Mg-OH was still much higher than the safety level. After the hydrophobic layer coated, the hemolysis rate of Mg-OH-(M/P) decreased to 2.03%, which is due to the improved corrosion resistance.

**Figure 11.**
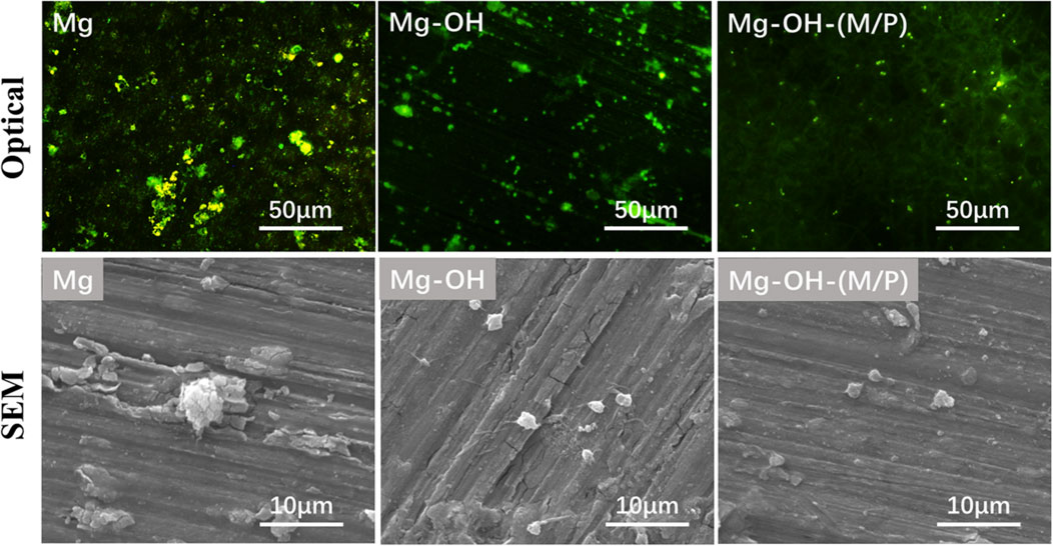
Fluorescence microscopy and SEM images SEM images of platelets adhered on the surface of Mg, Mg-OH and Mg-OH-(M/P) samples.

The fluorescence microscopy and SEM images of adherent platelets on the samples are shown in Fig. 11. The Mg-OH-(M/P) surface adhered fewest platelets according to the fluorescence images. The Mg-OH surface presented a larger amount of platelets than the Mg-OH-(M/P) surface, lower than the Mg surface. As the Mg is corroded, corrosion products appear on the surface, and it is difficult to distinguish platelets from the corrosion products. The shape of the platelets on samples can be observed from SEM images. The platelets adhering on Mg-OH-(M/P) showed less pseudopodia or activation than that on Mg-OH. The results showed that the Mg-OH-(M/P) with a superhydrophobic surface presented the best hemocompatibility among these samples.

### Cytocompatibility of ECs and SMCs

**Figure 12.**
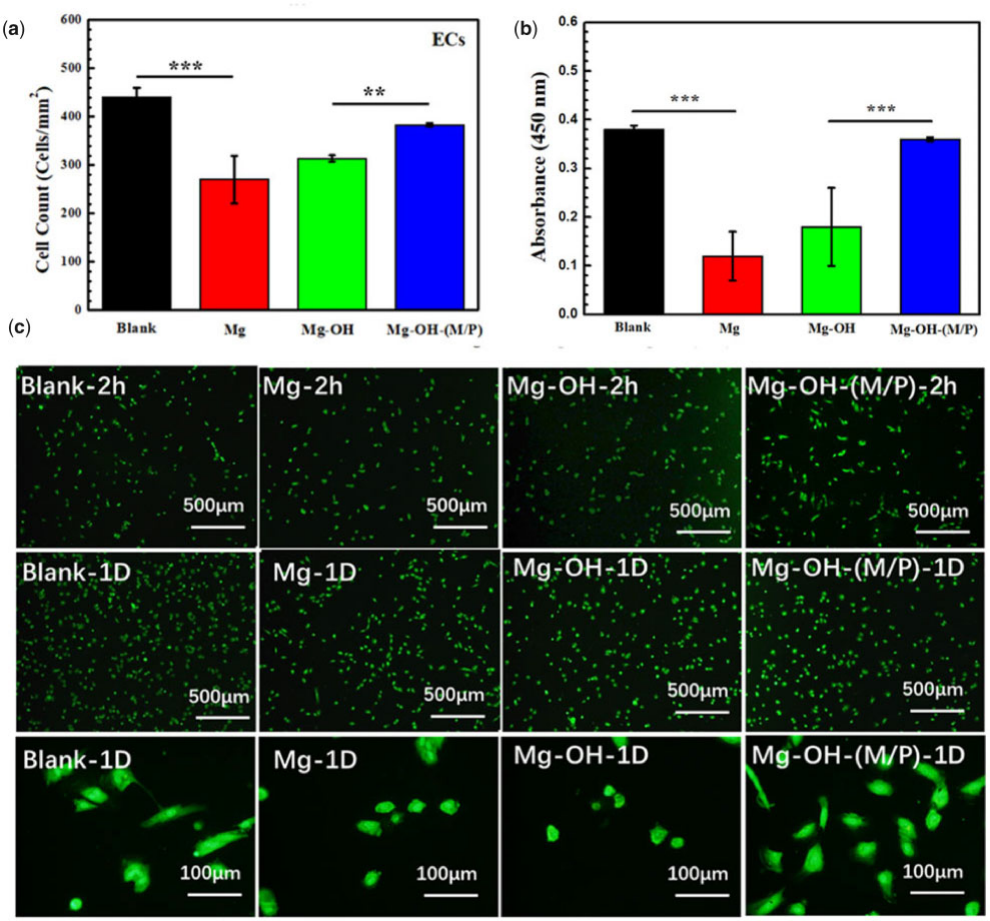
(**a**) Statistical counting of ECs on the plates (data presented as mean ± SD and analyzed using ANOVA, **P < 0.01, ***P < 0.001); (**b**) cell viability analyzed by Cell Counting Kit-8, CCK 8; (**c**) representative fluorescence images of ECs adhered on the plates after co-culturing with samples for 2 h and 1 day.

**Figure 13.**
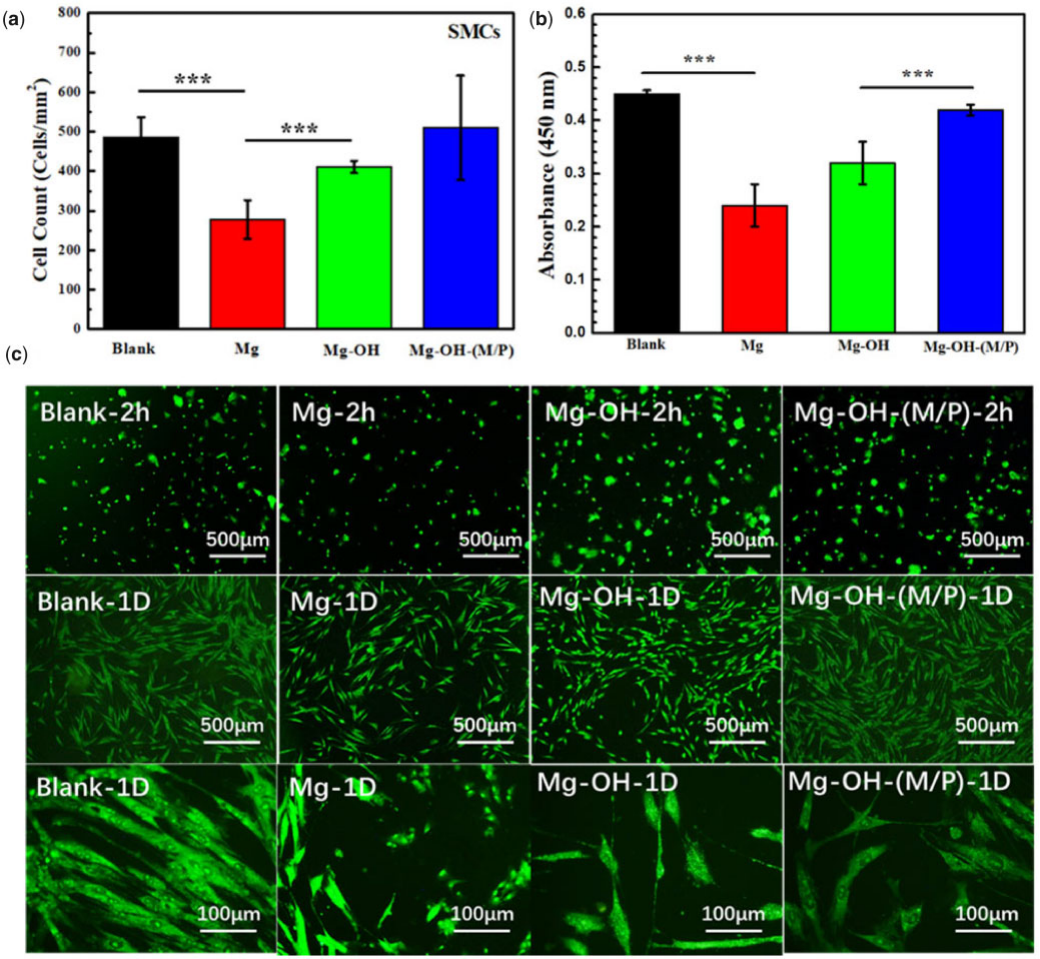
(**a**) Statistical counting of SMCs on the plates (data presented as mean ± SD and analyzed using ANOVA, ***P < 0.001); (**b**) cell viability analyzed by Cell Counting Kit-8, CCK 8; (**c**) representative fluorescence images of SMCs adhered on the plates after co-culturing with samples for 2 h and 1 day.

Since the cytocompatibility of blood vessel cells is a vital evaluation for vascular stent, the growth behavior of ECs and SMCs co-culture with samples was conducted in this study. The EC and SMC cell adhesion on the culture plate co-cultured with the samples after 2 h and 1 day were summarized in Figs 12 and 13. In order to obtain cell density and morphologies, the cell cytoskeleton was stained by rhodamine and detected by fluorescent microscopy. Figure 12a and b shows the number and viability of ECs indirectly contacted with samples for 1 day. There were obvious more cells indirectly contacted with the Mg-OH-(M/P) (383 ± 4 cells/mm^2^) than the Mg-OH (314 ± 6 cells/mm^2^) and the Mg (270 ± 49 cells/mm^2^) after 1 day incubation. And the viability around the Mg-OH-(M/P) was clearly larger than Mg and Mg-OH. The fluorescence microscopies of ECs co-cultured with samples for 2 h and 1 day are shown in Fig. 12c. The ECs grown indirectly contacted with the Mg-OH-(M/P) showed the characteristic cobblestone shape after 1 day. In comparison, cell shrinkage was observed indirectly contacted with the Mg and the Mg-OH. The hydrophobic layer on the Mg-OH-(M/P) had better corrosion resistance than other samples, showed lower hydrogen evolution and pH increases, which lead to better cytocompatibility of ECs.

The SMCs co-cultured with samples expressed the similar growth behavior to ECs. Figure 13a shows the number of adhered SMCs co-cultured with samples for 1 day. Due to the relatively fast degradation rate, the Mg displayed the lowest number of SMCs. After 1 day’s culture, the cell density of Mg-OH-(M/P) (510 ± 132 cells/mm^2^) was obviously higher than those of Mg (278 ± 49 cells/mm^2^) and Mg-OH (411 ± 16 cells/mm^2^). The SMCs ability results analyzed by CCK 8 are shown in Fig. 13b. There was obvious difference between Mg-OH-(M/P) and Mg, the result corresponded to the cell density. According to the fluorescence images in Fig. 13c, the adhered SMCs cultured around Mg presented an unnatural round shape with no elongation. The SMCs cultured around Mg-OH-(M/P) were more spread and elongated than those around Mg and Mg-OH for 2 h and 1 day’s culture. The layer on the Mg-OH-(M/P) reduced the corrosion rate of bare Mg, resulting in better growth state of SMCs. Since the modified samples were not grafted biomolecules for SMCs inhibition, the SMCs around the Mg-OH-(M/P) were not inhibited. However, the amine groups on Mg-OH-(M/P) can be used to graft related biomolecules to inhibit smooth muscle growth.

### Subcutaneous implantation

**Figure 14.**
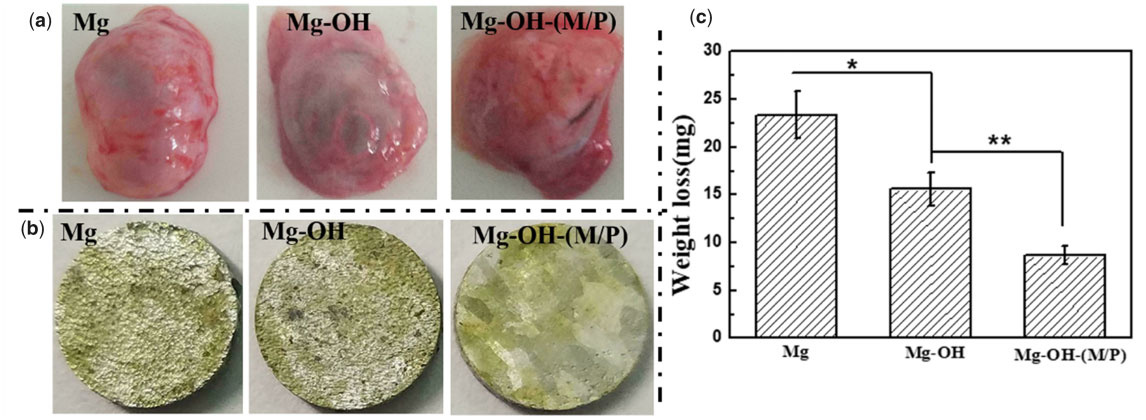
(**a**) Observation of the fibrous capsule, (**b**) corrosion morphology and (**c**) the weight loss of the samples after 30 days’ subcutaneous implantation.

The degradation behavior of the samples *in vivo* and the tissue response to the samples were evaluated by subcutaneous implantation in rats for 30 days, seen in Fig. 14. Figure 14a shows the morphology of the fibrous capsule around the samples, it is obvious that the fibrous capsule around the Mg is full of hydrogen, which indicates the fastest corrosion rate. The corrosion morphology of the implanted samples is shown in Fig. 14b, the untreated Mg and Mg-OH showed more serious degradation with corrosion pits emerging compared to the Mg-OH-(M/P). Figure 14c depicts the mass loss of Mg, Mg-OH and Mg-OH-(M/P) after implantation for 30 days. After 30 days implantation, the mass loss of Mg-OH-(M/P) was significantly lower compared to untreated Mg and Mg-OH. All the results above indicated that the Mg-OH-(M/P) showed the improved corrosion resistance compared to untreated Mg *in vivo*.

**Figure 15.**
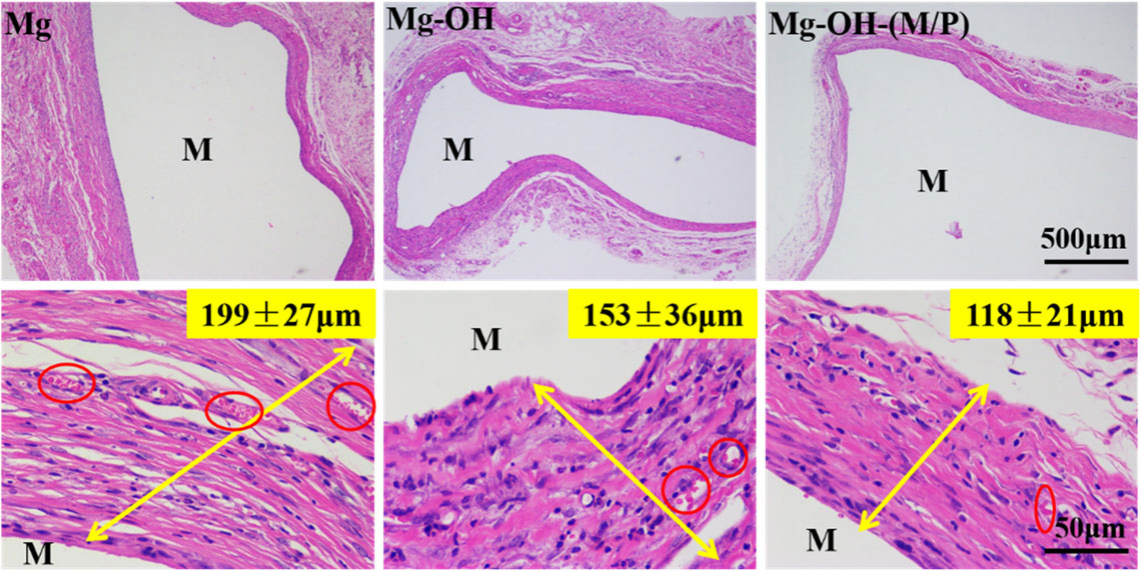
HE staining of subcutaneous tissues around samples after 30 days (M represents the implant site).

The histological evaluation of the tissue response to the implants is shown in Fig. 15. The thickness of fibrous encapsulation around the implants represents the extent of the tissue inflammation of the implants [52, 53]. After subcutaneous implantation for 30 days, a thick fibrous capsule (199 ± 27 μm) was observed around the Mg. The Mg-OH attenuated the fibrous capsule formation (153 ± 36 μm), indicating a milder inflammatory response. For Mg-OH-(M/P), there was even less granulation and a thinner fibrous capsule (118 ± 21 μm) formed at the interface due to the improved corrosion resistance. The red circle labeled region represents the capillary vessels, which indicated the inflammatory responses around the samples are evolving [52]. The capillary vessels around Mg-OH-(M/P) were smaller and not so dense compared to the other samples. The results indicated that the Mg-OH-(M/P) could mitigate the tissue response around the implantation site.

## Conclusion

In this study, a hydrophobic molecular layer was prepared by cyclic grafting of MDI and NH_2_-PDMS-NH_2_ on pure magnesium. The hydrophobic layer can provide corrosion resistance of substrate effectively. The static contact angle of water on the coated surface is 132.1°, which was due to the hydrophobic groups of the grafted MDI and NH_2_-PDMS-NH_2_. The hydrophobic surface significantly slows down the degradation rate of magnesium after being immersed in PBS for 210 h. The hydrophobic layer showed safe hemolysis rate and significantly reduced anti-platelet adhesion. The Mg-OH-(M/P) had better corrosion resistance, which led to better cytocompatibility of ECs and SMCs than bare Mg. Moreover, the coated sample improved corrosion resistance *in vivo* and mitigate the tissue response around the implants. Furthermore, the hydrophobic layer was rich of functional groups, which can be used for subsequent modification of biomolecules. This hydrophobic layer has great potential for constructing a multifunctional modified platform on the surface of biomedical magnesium stent.

## Funding

This work was supported by the National Natural Science Foundation of China (52101286), Sichuan Science and Technology Program (2022NSFSC2011) and Vanadium and Titanium Resource Comprehensive Utilization Key Laboratory of Sichuan Province (2018FTSZ29).

*Conflicts of interest statement*. None declared.
